# A New Way to Trace SARS-CoV-2 Variants Through Weighted Network Analysis of Frequency Trajectories of Mutations

**DOI:** 10.3389/fmicb.2022.859241

**Published:** 2022-03-16

**Authors:** Qiang Huang, Qiang Zhang, Paul W. Bible, Qiaoxing Liang, Fangfang Zheng, Ying Wang, Yuantao Hao, Yu Liu

**Affiliations:** ^1^Department of Medical Statistics and Epidemiology, School of Public Health, Sun Yat-sen University, Guangzhou, China; ^2^College of Computer, Chengdu University, Chengdu, China; ^3^College of Arts and Sciences, Marian University, Indianapolis, IN, United States; ^4^State Key Laboratory of Ophthalmology, Zhongshan Ophthalmic Center, Sun Yat-sen University, Guangzhou, China; ^5^School of Traditional Chinese Medicine Healthcare, Guangdong Food and Drug Vocational College, Guangzhou, China

**Keywords:** SARS-CoV-2, mutations, frequency trajectories, weighted network analysis, variant tracing

## Abstract

Early detection of SARS-CoV-2 variants enables timely tracking of clinically important strains in order to inform the public health response. Current subtype-based variant surveillance depending on prior subtype assignment according to lag features and their continuous risk assessment may delay this process. We proposed a weighted network framework to model the frequency trajectories of mutations (FTMs) for SARS-CoV-2 variant tracing, without requiring prior subtype assignment. This framework modularizes the FTMs and conglomerates synchronous FTMs together to represent the variants. It also generates module clusters to unveil the epidemic stages and their contemporaneous variants. Eventually, the module-based variants are assessed by phylogenetic tree through sub-sampling to facilitate communication and control of the epidemic. This process was benchmarked using worldwide GISAID data, which not only demonstrated all the methodology features but also showed the module-based variant identification had highly specific and sensitive mapping with the global phylogenetic tree. When applying this process to regional data like India and South Africa for SARS-CoV-2 variant surveillance, the approach clearly elucidated the national dispersal history of the viral variants and their co-circulation pattern, and provided much earlier warning of Beta (B.1.351), Delta (B.1.617.2), and Omicron (B.1.1.529). In summary, our work showed that the weighted network modeling of FTMs enables us to rapidly and easily track down SARS-CoV-2 variants overcoming prior viral subtyping with lag features, accelerating the understanding and surveillance of COVID-19.

## Introduction

The severe acute respiratory syndrome-related coronavirus 2 (SARS-CoV-2) causing coronavirus disease 2019 (COVID-19) has been running rampant all over the world since December 2019. The current pandemic has triggered an unprecedented scale of whole-genome sequencing and sharing of the virus’s genome. Surveillance of SARS-CoV-2 variants using sequence data provides insight into disease virulence, pathogenesis, host range or immune escape, as well as the effectiveness of SARS-CoV-2 diagnostics and therapeutics (Grubaugh et al., 2021; Tegally et al., 2021). Viral subtyping methods such as GISAID (Han et al., 2019), Pangolin (Rambaut et al., 2020) and CMM (Qin et al., 2021) have greatly aided this process. Designating a subtype (e.g., lineage) for each genome according to predetermined genetic features (e.g., mutations) followed by continuous risk assessment of these subtypes serves to identify clinically important emerging variants. However, subtype assignment depends on lag features that may delay the detection of newly emerging variants or the descendants of circulating variants. In addition, a too detailed subtyping (e.g., Pangolin) of the SARS-CoV-2 population has resulted in excess burden on risk monitoring while a rough categorization (e.g., GISAID) delays the detection and communication of dangerous variants (Oude Munnink et al., 2021; Qin et al., 2021; Tang et al., 2021).

It is well known that new SARS-CoV-2 variants with their specific mutation features gradually dominate through spatial and temporal expansion (Mascola et al., 2021). The frequencies of different mutations throughout the viral genome can now be tracked over time with high resolution and reliability. Mutations with synchronous frequency trajectories are likely to define a variant or a group of variants (Zhao et al., 2020; Bernasconi et al., 2021; Qin et al., 2021). Thereby, the frequency trajectories of mutations (FTMs) contain information that could allow very sensitive detection of prevalent mutations highlighting important variants, e.g., variants under investigation (VUI) or variants of concern (VOC). Leveraging FTMs to develop new analytics will allow truly real-time surveillance of SARS-CoV-2 variants and improve the lead time for public health interventions.

In this paper, we developed a module-based variant surveillance method that enables real-time tracking of historical and circulating SARS-CoV-2 variants without designating their subtypes in advance allowing newly emerging variants or the descendants of circulating variants to be tracked earlier. This method views mutations represented by FTMs as nodes of a network and describes their relationships using network connections. We found that closely connected nodes in the network forming a biologically meaningful module indicate a potential variant, and module clusters indicate potential contemporaneous variants. We demonstrate the FTM network construction and interpretation through analysis of worldwide data of SARS-CoV-2 genomes and validate its variant surveillance capability *via* tracking the variants circulating in two COVID-19 hotspots, India and South Africa.

## Materials and Methods

**FIGURE 1:**
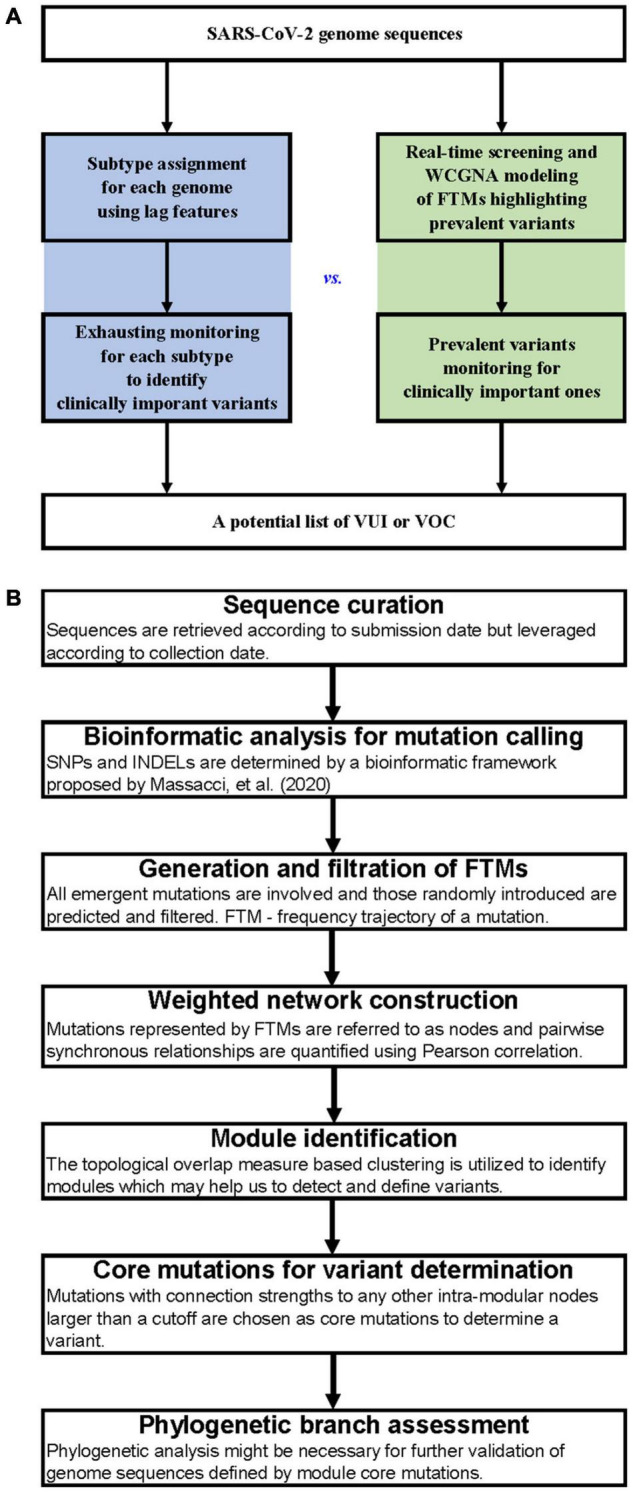
Workflows for SARS-CoV-2 variant surveillance. **(A)** Workflow comparison between subtype-based and FTM-based variant surveillance methods. **(B)** Outline of a weighted network framework for variant surveillance using FTMs.

A comparison of the workflows between subtype-based and FTM-based variant surveillance methods has been shown in Figure 1A. The outline of our FTM-based SARS-CoV-2 variant identification framework using weighted network modeling is shown in Figure 1B. This framework uses FTMs as an input and is comprised of the following main steps: sequence curation, mutation calling, calculation, and filtering of FTMs, network construction, variant identification and determination using core mutations, and variant validation. We used the worldwide data and the pandemic variants (Supplementary Table 1) as a benchmark and further illustrate the surveillance features of our method using regional data from India and South Africa. Below, we focus on the delineation of each step.

### Data Curation

SARS-CoV-2 genomes were retrieved from GISAID database (Shu and McCauley, 2017). Only viruses from human submitted before 2021-11-30 with sample collection date between 2020-01-05 and 2021-11-27 were extracted, filtering sequences with flags, “complete sample collection date”, “complete genome” (genome length > 29,000 bp) and “low coverage excluded” (exclude genomes with > 5% Ns). Consequently, a total of 5,043,950 genomes were collected. Because significant sampling date errors were found in metadata of some genomes (Supplementary Figure 1), they were firstly excluded from downstream analysis according to their mutation numbers (see below).

### Bioinformatic Analysis for Mutation Calling

Whole genome genetic variations, including single nucleotide polymorphisms (SNPs) and insertions/deletions (INDELs), were determined and annotated using a bioinformatic framework proposed by Massacci et al. (2020) with Wuhan-Hu-1 (GenBank NC_045512.2) (Wu et al., 2020) as the reference. In summary, the viral sequences were first aligned against the reference using the nucmer command with default settings except requiring only the forward matching of the query sequences (–forward), provided by the MUMmer package (version 3.23) (Marcais et al., 2018). The generated delta encoded alignment files were then parsed by the show-snps command to produce a catalog of all SNPs and INDELs. Show-snps outputs were summarized and translated to proteins using a R script adapted from Mercatelli and Giorgi (2020). Eventually, an annotated list including 186,399,389 mutational events was exported. The number of mutational events for each study sample was firstly calculated. Since high mutation numbers are not likely to appear in the early stage of the COVID-19 pandemic (Supplementary Figure 1), we excluded genomes with mutation numbers far beyond other samples collected in the same month, where the cutoffs were set to be the average plus 5 standard deviations.

### Calculation and Filtration of Frequency Trajectories of Mutations

Mutations present at least once across all genomes were extracted and their frequency time series were generated according to calendar weeks of sampling. Specifically, a mutation frequency, denoted by *y*_st_, at a sampling week *t* on a specific site *s* was calculated as the fraction of genomes with the mutation of all genomes sampled at that week. Then the frequency trajectory of a mutation *s*(1 = *s* = *S*) can be denoted as


(1) $${\text{y}}_{s}=\left\{y_{s\,t}:1\leq t\leq T\right\},$$

where *t* denotes the week number and *t* = 1 represents the first complete calendar week of 2020 (from January 5 to 11, 2020). When aggregating the mutation events for each mutation site, a large amount of multi-directional mutations (e.g., C→T and C→G) were detected (Supplementary Figure 2). All possible mutation directions were considered in our study to allow the distinction of different variant branches (e.g., G23012A for B.1.351 and G23012C for B.1.617.1) and to avoid erroneous clustering in the network construction due to missing mutation directions.

A myriad of mutations (i.e., large *S*) were detected across the viral genome but most were less informative with the temporal frequency pattern of fluctuating near zero (Supplementary Figure 3). Therefore, FTMs with all mutation frequencies less than a threshold [e.g., 1%, a threshold above which a mutation is considered fixed in a natural population (Wong et al., 2003)] were first excluded. For worldwide data, 1,178 (1.4%) were kept after this filtration and the majority of these FTMs maintained a frequency of ≥1% only for a limited period, as described by Chiara et al. (2021). To facilitate the demonstration of the methodology features, a hierarchical clustering analysis using Ward’s method (Ward, 1963) was additionally applied to group and exclude them before investigating the temporal clustering patterns.

### Weighted Network Construction

In the network model, nodes correspond to mutations, or more precisely to scaled FTMs with


(2) $${\text{y}}_{s}^{\prime }=\frac{{\text{y}}_{s}-m\,e\,a\,n\,\left({\text{y}}_{s}\right)}{\sqrt{v\,a\,r\,\left({\text{y}}_{s}\right)}}$$

where $m\,e\,a\,n\,\left({\text{y}}_{s}\right)=\frac{1}{T}\,\sum_{t}y_{s\,t}$ and $v\,a\,r\,\left({\text{y}}_{s}\right)=\frac{1}{T-1}\,\sum_{t}{\left[y_{s\,t}-m\,e\,a\,n\,\left({\text{y}}_{s}\right)\right]}^{2}$. The edges between mutations are determined by the pairwise Pearson correlations between FTMs. Then two FTMs will have a correlation coefficient close to 1 if they are synchronous, and non-synchronous relationships will deviate from 1. The connection strength between mutation *i* and *j* were quantified with an adjacency score using a power function (Horvath, 2011),


(3) $$A_{i\,j}={\left(0.5+0.5\cdot c\,o\,r\,r\,\left({\text{y}}_{i}^{\prime },{\text{y}}_{j}^{\prime }\right)\right)}^{\beta },$$

where$c\,o\,r\,r\,\left({\text{y}}_{i}^{\prime },{\text{y}}_{j}^{\prime }\right)$is the Pearson correlation coefficient between ${\text{y}}_{i}^{\prime }$ and ${\text{y}}_{j}^{\prime }$. The transformation in the parentheses is applied to map the correlations onto the interval [0, 1] to satisfy the requirement of an adjacency matrix and the exponential transformation with β≥1 is used to emphasize strong correlations at the expense of weak correlations. This leads to a weighted network and β is determined based on the scale-free topology criterion (Zhang and Horvath, 2005).

The network connectivity (*k_s*) of the *s*th mutation is the sum of the connection strengths with the other mutations, *k*_*s*_ = ∑_*i*≠*s*_*A*_*si*_. The summation performed over all mutations in a particular module is the intra-modular connectivity (*k*_s,intra_).

### Network Module Identification

In weighted networks, modules are subsets of mutations which are tightly connected. Identifying these modules facilitates rapid identification and designation of a variant. Since the adjacency between two nodes cannot reflect their connectivity with other intra- or inter-modular nodes, we use a topological overlap measure (TOM) instead. The topological overlap is defined by:


(4) $$T\,O\,M_{i\,j}=\left\{ \begin{array}{c} \frac{\sum_{l\neq i,j}A_{i\,l}\,A_{l\,j}+A_{i\,j}}{\min\left(k_{i},k_{j}\right)+1-A_{i\,j}}\,\text{i}\neq \text{ j}\\ 1\;i=j \end{array}\right.$$

where ∑_*l*≠*i*,*j*_*A*_*il*_*A*_*lj*_ quantifies the indirect connection strengths between *i* and *j* through their shared neighbors and the denominator serves as a normalization factor. The topological overlap between mutation *i* and *j* reflects their relative interconnectedness as mediated through other mutation nodes (Yip and Horvath, 2007). Module identification was done using the TOM-based dissimilarity matrix diss*TOM* = (1−*TOM*_ij_) coupled with average linkage hierarchical clustering. Modules corresponded to branches of the resulting hierarchical clustering tree. We used a dynamic cut-tree algorithm to determine the branches (Langfelder et al., 2008). All of these were realized with the R WGCNA package (Langfelder and Horvath, 2008).

To intuitively display the relationship between nodes of the weighted network, the topological overlap matrix was partitioned by different cutoffs (e.g., 0.1 or higher) and visualized using the R igraph package (Csárdi and Nepusz, 2006). To distinguish between modules, each module was designated with a visually friendly color.

### Core Mutations for Variant Determination

According to our hypothesis, modules in our network are expected to be sets of synchronous FTMs that represent variants. Emerging variants develop mutations quickly, but they are characterized by a highly correlated set of characteristic mutations. These characteristic mutations form densely connected intra-modular sub-networks. These sub-networks represent the “core” of a module and are detected using a high-pass adjacency score threshold. The threshold value is determined empirically by mapping benchmark modules to the global phylogeny (see below) with statistical evaluation of specificity and sensitivity. The historical classification and nomenclature for these variants were extracted from the GISAID metadata.

### Phylogenetic Assessment of Detected Variants

We assessed variants determined by our module “core” mutations against a global reference dataset provided by GISAID using the pipeline proposed by Nextstrain (Hadfield et al., 2018). First, the metadata of the global SARS-CoV-2 phylogenic tree, with 4,506,129 high quality genomes created on December 24, 2021, were retrieved from the GISAID database. A subsample randomly selected from these data was used for the skeleton construction of global SARS-CoV-2 phylogenic tree. Second, the module genomes determined by the module “core” mutations were extracted. Specifically, the pandemic module genomes pointing to S, V, G, GH, GV, GR, GRY, and GK were directly taken from the global reference dataset to show the consistency with the skeleton tree. Other module genomes were extracted from the source data but down-sampling was introduced if the number exceeded 200. Then, the pipeline successively performs an alignment of genomes in MAFFT (Katoh and Standley, 2013), phylogenetic inference in IQ-Tree (Minh et al., 2020), tree dating and ancestral state construction and annotation. The phylogenetic trees were visualized using the R ggtree package (Yu et al., 2018).

## Results

### Variant-Specific Frequency Trajectories of Mutations Present Synchronous Temporal Changes

**FIGURE 2:**
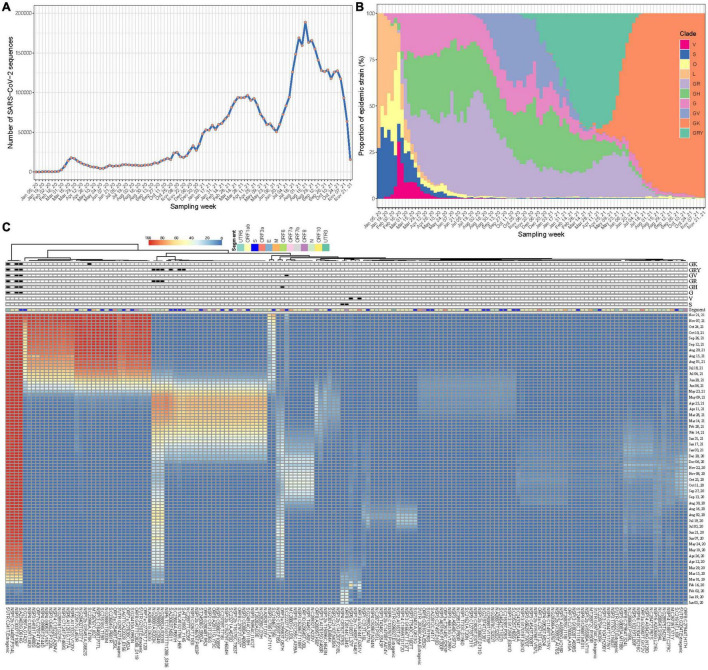
Synchronous temporal changes between variant-specific FTMs and variant prevalence. **(A)** Weekly distribution of SARS-CoV-2 genome sequences according to sampling time. **(B)** Time course of major variant distribution in collected sequences. **(C)** A Wald’s linkage hierarchical cluster tree of frequency trajectories of mutations. One hundred and fifty-eight mutations passing filtration were analyzed, annotated and displayed. Variant-specific mutations were flagged.

A total of 5,043,950 SARS-CoV-2 sequences during our study period were retrieved. After excluding those with probable sampling date error, 5,042,287 (>99.9%) were eventually included. These viral sequences have been accumulating over time at an unprecedented speed, from a few to hundreds of thousands a week according to their sampling time (Figure 2A). Changes in the prevalence of the SARS-CoV-2 variants over time have been imprinted through these sequences (Figure 2B). Using Wuhan-Hu-1 genome (NC_045512.2) as the reference, 186,253,697 mutation events were detected at 29,825 nucleotide sites, including 28,972 (97.1%) sites with 2 or more mutation directions (Supplementary Figure 2). The time series plots of FTMs showed that majority of them had very low occurrence rate over time (Supplementary Figure 3), indicating a high chance of random or unstable mutations, or even sequencing artifacts. A few mutations with synchronous temporal changes (e.g., C241T, C3037T, C14408T and A23403G) were also observed.

To show the association of epidemic variants and genetic variations of SARS-CoV-2 across time, a clustering process using Ward’s method was done for the FTMs. Due to ultra-high analytic dimensionality, the cluster having randomly fluctuated series was firstly identified and excluded (see section “Materials and Methods”). In consequence, 158 time series were left. The clustering analysis showed that mutations with consistent temporal change patterns were clustered together and some of these clusters were clearly linked to variant features (Figure 2C). This suggests that frequency trajectories of variant-specific mutations can be used for identifying and tracking variants. Moreover, there exist other mutation trajectories within each cluster having synchronous temporal changes (Figure 2C), which indicates the availability of more information that can be used to trace the same variant.

### Identification of Variants Using the Weighted Network

The weighted network workflow for SARS-CoV-2 variant tracking has been summarized in Figure 1B and detailed in section “Materials and Methods”. Briefly, the Pearson correlation coefficient is calculated for all pair-wise comparisons of the scaled FTMs across the viral genome. This correlation matrix is then transformed into a matrix of connection strengths using a power function (connection strength = (0.5 + 0.5 × correlation)^β^). Mutations with similar patterns of connection strengths are speculated to form network modules while each node represents an FTM-related mutation. Topological overlaps are used to assess the similarity of the synchronous relationship of two FTMs with all the other FTMs in the network. Modules with high topological overlaps are detected using average linkage hierarchical clustering coupled with a dynamic tree-cutting algorithm. Each module is analyzed separately to identify “core” mutations for variant determination.

**FIGURE 3:**
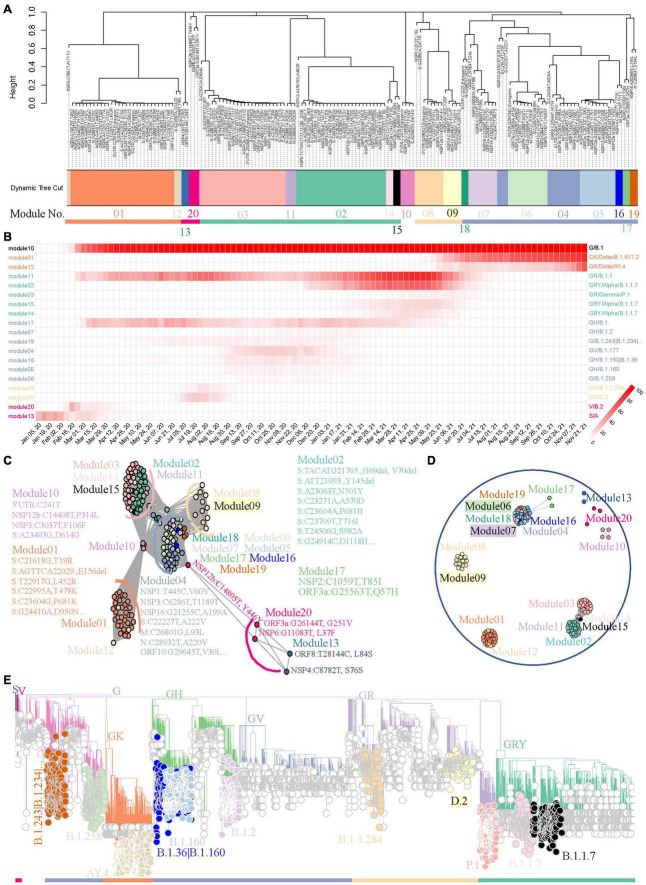
A benchmark to use weighted network framework for identification of worldwide pandemic variants. **(A)** Clustering dendrogram of 158 FTMs from GISAID worldwide data. The module numbers are labeled and module clusters are highlighted with different colors. **(B)** The heatmap of module-based variant prevalence. The variants were determined by core mutations within each module. The modules were reordered and colored according to their module clusters and time course. **(C)** Network graph with topology overlap values > 10^– 3^ to show the relationship between nodes and modules of the weighted network. **(D)** Network graph with topology overlap values > 0.1. **(E)** Phylogenic evaluation of detected worldwide pandemic variants. Time-resolved maximum clade credibility phylogeny is shown and identified variants are highlighted and annotated with visually friendly colors.

We used the 158 most frequent mutations from worldwide data for module detection and variant identification to show the capability of the method to track variants using a weighted network. This may lead to some information loss about the endemic variants, but we will illustrate later that this workflow will be more sensitive when it is applied to regional data. As showed in Figure 3A, FTMs were grouped into 20 distinct modules with 5 module clusters. Most modules (19/20, 95.0%) point to well-defined variants supported by the module genomes, which were identified from the viral population through the module core mutations (Supplementary Table 2). More precisely, 8 modules were clearly linked to global epidemic clades (S, V, G, GH, GV, GR, GRY, and GK) and 11 were identified as variants or sub-variants causing tens of thousands of COVID-19 cases, including two sub-variants of GRY that were not assigned Pangolin lineages. All the identification showed a very high specificity approaching 100% and a high sensitivity exceeding 70% when using the global phylogeny as a reference with an adjacency cutoff 0.7, an appropriate compromise between area under the receiver-operating characteristic curve and module-based variant discovery (Supplementary Table 3). Another module showed low connection strengths (<0.4) between nodes indicating asynchronous FTMs; thus, it was ignored. In addition, the time course prevalence of the module-based variants suggested that the 5 module clusters represented the five worldwide epidemic stages until the late of November, 2021, with co-circulation of multiple major variants defined by intra-cluster modules during each period (Figure 3B).

Network graphs were used to further demonstrate the relationships among nodes within a module as well as to inspect how any module is related to the rest of the network and how closely any two modules are related. The continuous network topology was dichotomized by different cutoffs, and modules were individually colored. These network graphs highlighted our FTM-based weighted network conglomerated variant-specific mutations as modules with contemporaneous variants forming module clusters. First, mutations pointing to the same variant were clustered together to form closely connected modules (Figure 3C). Second, the modules pointing to cotemporaneous variants were likely to be connected to each other (Figure 3C). Third, with the increasing cutoff, linkages were broken in turn, first between module clusters and then between intra-modular nodes (Figure 3D and Supplementary Figure 4). All of these method features provided us with fresh insights to track down the historical, current, or emerging variants.

### Validation Using Phylogenic Analysis

Variants determined by core mutations (Supplementary Table 2) were evaluated using phylogenic analysis. Data randomly sampled from the global SARS-CoV-2 phylogenic tree of the GISAID repository were used to establish the phylogenic skeleton (Supplementary Figure 5A). Genomes with module “core” mutations of S, V, G, GH, GR, GRY, and GK in the skeleton showed almost perfect consistency with the expectation (Supplementary Figure 5B). Samples with other module “core” mutations were selected from the source data, an updated phylogenetic tree was generated, and nodes were colored by their modules. As shown in Figure 3E, module “core” mutations detected by our weighted network successfully identified their lineages.

### Workflow Application for Variant Surveillance in India and South Africa

**FIGURE 4:**
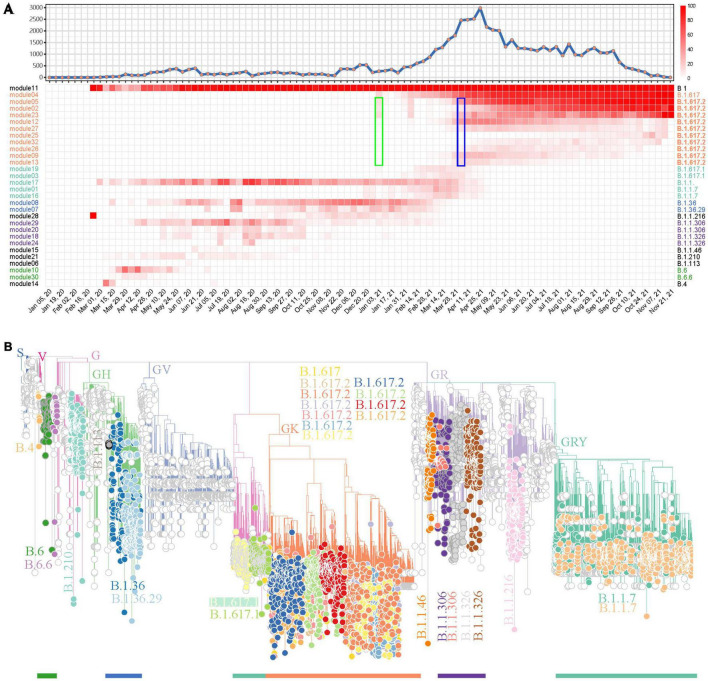
A demonstration to identify SARS-CoV-2 variants prevalent in India using the weighted network. **(A)** Weekly distribution of SARS-CoV-2 genome sequences according to sampling time (top) and the heatmap of module-based variant prevalence (bottom). Core mutations within each module were used to define the variants. The modules were reordered and colored according to their module clusters and time course. The weeks when Delta (B.1.617.2) was identified as a prevalent variant by network model (green) or reported as a VUI by WHO (blue) are highlighted by rectangles. **(B)** Phylogenic evaluation of detected endemic SARS-CoV-2 variants in South Africa. The detected variants are highlighted and annotated with visually friendly colors.

After showing the capability of weighted network analysis of FTMs in module-based variant identification, we applied this workflow for SARS-CoV-2 variant surveillance in regional data and further tested its efficacy. All 59,069 SARS-CoV-2 genomes in the study period from India were first included. Since the genome numbers in the sampling weeks showed a high fluctuation (Figure 4A), from zero to several thousand, we only kept mutations that have occurred in 10% or more of genomes with occurrences > 10 in at least one sampling week. This resulted in 165 FTMs left for the weighted network construction. Following the automatic parameter selection and clustering process, these mutations were grouped into 33 modules among which 30 (30/33, 90.9%) had sets of mutations with strong synchronous FTMs (Supplementary Table 4). Five module clusters were detected in this process (Supplementary Figure 6). According to this module clustering feature, the heatmap of module-based variant prevalence clearly showed the SARS-CoV-2 epidemic in India by November 2021 could be divided into at least five stages, with the major variants during each stage determined by the module core mutations (Figure 4A). Phylogenetic assessment through a module-based sampling confirmed the results of network analysis and showed the modules corresponded to B.1.617.2 (Delta), B.1.617.1 (Kappa), B.1.1.7 (Alpha), B.1.36, B.1.1.306, B.1.1.326 or their sub-variants (Figure 4B). It is noteworthy that the weighted network would provide much earlier warning of Delta (B.1.617.2) than the date it was reported as VUI by WHO (January 3 2021 vs. April 4 2021, Figure 4A), if the time delay between sample collection, sequencing and analysis could be sufficiently overcome. In addition, the phylogenetic tree suggested that the network analysis detected multiple descendants of the major SARS-CoV-2 variants previously or currently circulating in India. Specifically, four primary descendent variants of B.1.617.2 (Figure 4B), which continued circulating as a dominant lineage in India until the end of November 2021, were tracked down. In contrast, CMM classified this variant to G3.14.1 with no subtype surveillance and Pangolin gave various subtypes of this variant (Supplementary Table 5).

**FIGURE 5:**
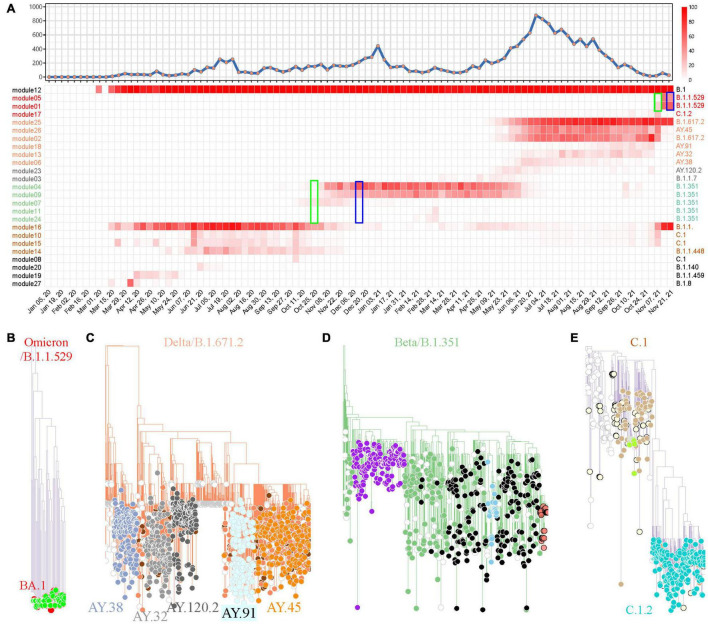
SARS-CoV-2 variant surveillance in South Africa using the weighted network. **(A)** Weekly distribution of SARS-CoV-2 genome sequences according to sampling time (top) and the heatmap of module-based variant prevalence (bottom). Core mutations within each module were used to define the variants. The modules were reordered and colored according to their module clusters and time course. The weeks when Omicron (B.1.1.529) and Beta (B.1.351) were identified as prevalent variants by network model (green) or reported as VUI or VOC by WHO (blue) are highlighted by rectangles. **(B–E)** Phylogenic evaluation of every major SARS-CoV-2 variant detected in South Africa, including Omicron **(B)**, Delta **(C)**, Beta **(D)** and C.1 **(E)**. The module-based variants having consistent classification with Pangolin lineages are labeled.

The same pipeline was applied in South Africa for SARS-CoV-2 variant tracking. The weighted network modeling for FTMs generated by the total available 17,778 SARS-CoV-2 genomes showed viral population in South Africa has gone through four prevalent stages with variant cluster pattern (Figure 5A), including a rapid surging of suspected variants with numerous spike protein mutations detected since November 7, 2021 (Supplementary Table 6 and Supplementary Figure 7). The newly circulating variants seemed to split from module 25 with mutation C10029T and C22995A according to the prevalence rate. Phylogenetic analysis using module-based sampling data showed that the dominant variants at the four stages were B.1.1.529 (Omicron), B.1.617.2, B.1.351, and C.1, respectively, from near to far (Figures 5B–E). The descendants of these variants were also tracked down by the weighted network, having consistent but more dedicated subtypes compared with Pangolin classification and more detailed than CMM grouping (Supplementary Table 7).

## Discussion

Scientists are keeping their eyes open for the mutating SARS-CoV-2 virus and making every effort to detect, investigate, and monitor clinically important variants (Chakraborty et al., 2021; Grubaugh et al., 2021; Mascola et al., 2021). In this study, we proposed a module-based variant surveillance framework through weighted network modeling of FTMs, enabling us to rapidly gain insights into the time-scaled dispersal history of SARS-CoV-2 variants without requiring prior lineage assignment of each viral sequence (Figure 1A). This framework modularizes the FTMs, with synchronous FTMs conglomerating together to represent the variants and module clusters reflecting contemporaneous variants (Figure 3C). The module-based variants are assessed by phylogenetic tree through sub-sampling to facilitate communication and control of the epidemic.

The ad hoc viral classification may delay the detection of newly emerging variants or their descendants. Viral subtyping followed by their characterization, prevalence monitoring and risk assessment is continuing to be used in SARS-CoV-2 variant surveillance (World Health Organization [WHO], 2021). Either phylogenetic-tree-based partition of GISAID (Han et al., 2019), Nextstrain (Hadfield et al., 2018) and Pangolin (Rambaut et al., 2020), or genetic-feature-based grouping of CMMs (Qin et al., 2021) and ISMs (Zhao et al., 2020), captured viral subtype features according to historical data, resulting in lag signals of classification, and then false subtyping at the early stage of their emergence delayed the public health response. Our module-based variant surveillance would have provided much earlier warning about newly surging variants of B.1.617.2 in India (Figure 4A) and B.1.351/B.1.1.529 in South Africa (Figure 5A) prior to their announced VUI/VOC dates by WHO.

Our investigation also reveals other advantages of module-based variant monitoring. First, the surveillance system will automatically divide the whole epidemic period into multiple stages and detect variant co-circulation pattern during each stage (Figures 3B, 4A, 5A). This may give an important insight into viral evolution (Kostaki et al., 2021). Second, the methodology provides variant surveillance at moderate resolution, facilitating an overview of epidemic variants. Our framework focuses on the tracking of prevalent variants rather than comprehensive surveillance. In spite of a rough filtration process, the benchmark analysis using worldwide data tracked down all the major pandemic variants and some regionally epidemic variants (Figure 3E). National level analysis in India and South Africa further demonstrated that this approach not only provided a variant profile (Figures 4B, 5B–E) consistent with previous studies (Singh et al., 2021; Tegally et al., 2021), but also gave more detailed variant monitoring than CMM. The weighted network analysis also provided a much more enriched variant investigation than Pangolin (Supplementary Tables 5, 7), which were confirmed by previous reports. Third, our framework allows insertion, deletion and recombination events to be included. This highly extends the surveillance because current variant monitoring mainly involves substitution events (Zhao et al., 2020; Qin et al., 2021) and poses a great challenge in phylogenetic inference (Liu et al., 2021).

Our approach can be an alternative method for rapid investigation and early detection of prevalent variants to facilitate regional SARS-CoV-2 genomic surveillance. An efficient variant surveillance is firstly dependent on the timely availability of viral genomes (Kalia et al., 2021). To compensate and minimize the time delay between sample collection and submission, surveillance activities at national and sub-national levels, where first hand data are actually acquired, are highly recommended (World Health Organization [WHO], 2021). Meanwhile, simple surveillance systems, especially employing time-based analysis of SARS-CoV-2 mutations, are developed to assist in the identification of candidate variants of clinical importance. Nevertheless, most of them focus on trend survey of viral mutations (Wada et al., 2020; Showers et al., 2022) or their phenetic clustering (Yang et al., 2020; Chiara et al., 2021) but not real variant monitoring. Based on similar motivation, Bernasconi et al. (2021) applied standard time-series clustering to group 1-month-long FTMs for detection of all SARS-CoV-2 variants at national level. Due to the segmenting and complete analysis of FTMs, they have to face the challenge of handling the discrepancies between cluster features of the same variants, especially when these variants are new and not included in the lineage dictionary. Our module-based variant monitoring overcomes these difficulties by concentrating on high-frequency FTMs for prevalent variant identification.

Some limitations are also acknowledged. First, the mutation modules detected by our workflow may not represent a nominated lineage, but the analysis offers perceptive insights into novel variants which could be causing more transmission. Second, the independence between FTMs were assumed in the analysis. This might not be true especially for multiple direction mutations at the same nucleotide sites. However, as we can see in our analysis, the assumption may not highly influence our results. Lastly, the threshold value of FTM filtration is empirically chosen. This may result in the loss of less frequent variants. We believe it is a trade-off between detectability and discriminability in variant monitoring. When more samples are available and the cutoff is thought to be too big, analysis at a higher spatial resolution is recommended.

In summary, an efficient and easy-to-use weighted network framework was proposed for SARS-CoV-2 variants tracing that could help to accelerate the understanding, surveillance, and control of the emerging viral variants.

## Data Availability Statement

The original contributions presented in the study are included in the article/Supplementary Material, further inquiries can be directed to the corresponding author/s.

## Author Contributions

YL and YH conceived, designed, and supervised the project. QL and FZ collected the data. QH, QZ, YW, and YL performed computations, analyzed the results, and drafted the manuscript. PB and YH provided critical revision for important intellectual content. All authors contributed to the article and approved the submitted version.

## Conflict of Interest

The authors declare that the research was conducted in the absence of any commercial or financial relationships that could be construed as a potential conflict of interest.

## Publisher’s Note

All claims expressed in this article are solely those of the authors and do not necessarily represent those of their affiliated organizations, or those of the publisher, the editors and the reviewers. Any product that may be evaluated in this article, or claim that may be made by its manufacturer, is not guaranteed or endorsed by the publisher.

## Supplementary Material

Supplementary Figure 1 Frequency distribution of mutational number of each SARS-CoV-2 genome at each sampling week.

Supplementary Figure 2 Frequency distribution of number of mutation directions at each mutation sites.

Supplementary Figure 3 Frequency trajectories of mutations by SARS-CoV-2 genome regions. UTR, Untranslated region; NSP, non-structural protein; S, Spike protein; ORF, Open reading frame; M, Membrane protein; N, Nucleocapsid protein; E, Envelope protein.

Supplementary Figure 4 Network graphs with different topological overlap cutoffs for identification of worldwide pandemic variants.

Supplementary Figure 5 The global SARS-CoV-2 phylogenic skeleton. **(A)** The SARS-CoV-2 phylogenic skeleton generated by the Nextstrain pipeline based on a random sample of the global phylogenic tree from the GISAID database, with the edges colored by the GISAID clade nomenclature system. **(B)** Comparison of the genome classification consistency between the expectation and those determined by the “core” mutations.

Supplementary Figure 6 Clustering dendrogram of 165 FTMs from India, with dissimilarity based on topological overlap. The module numbers were labeled and module clusters were highlighted with different colors.

Supplementary Figure 7 Clustering dendrogram of 223 FTMs from South Africa, with dissimilarity based on topological overlap. The module numbers were labeled and module clusters were highlighted with different colors.
