# Supplementary figures and images for: A New Way to Trace SARS-CoV-2 Variants Through Weighted Network Analysis of Frequency Trajectories of Mutations
Source: Front Microbiol. 2022 Mar 16;13:859241. doi: 10.3389/fmicb.2022.859241 (PMC8966897; doi:10.3389/fmicb.2022.859241)

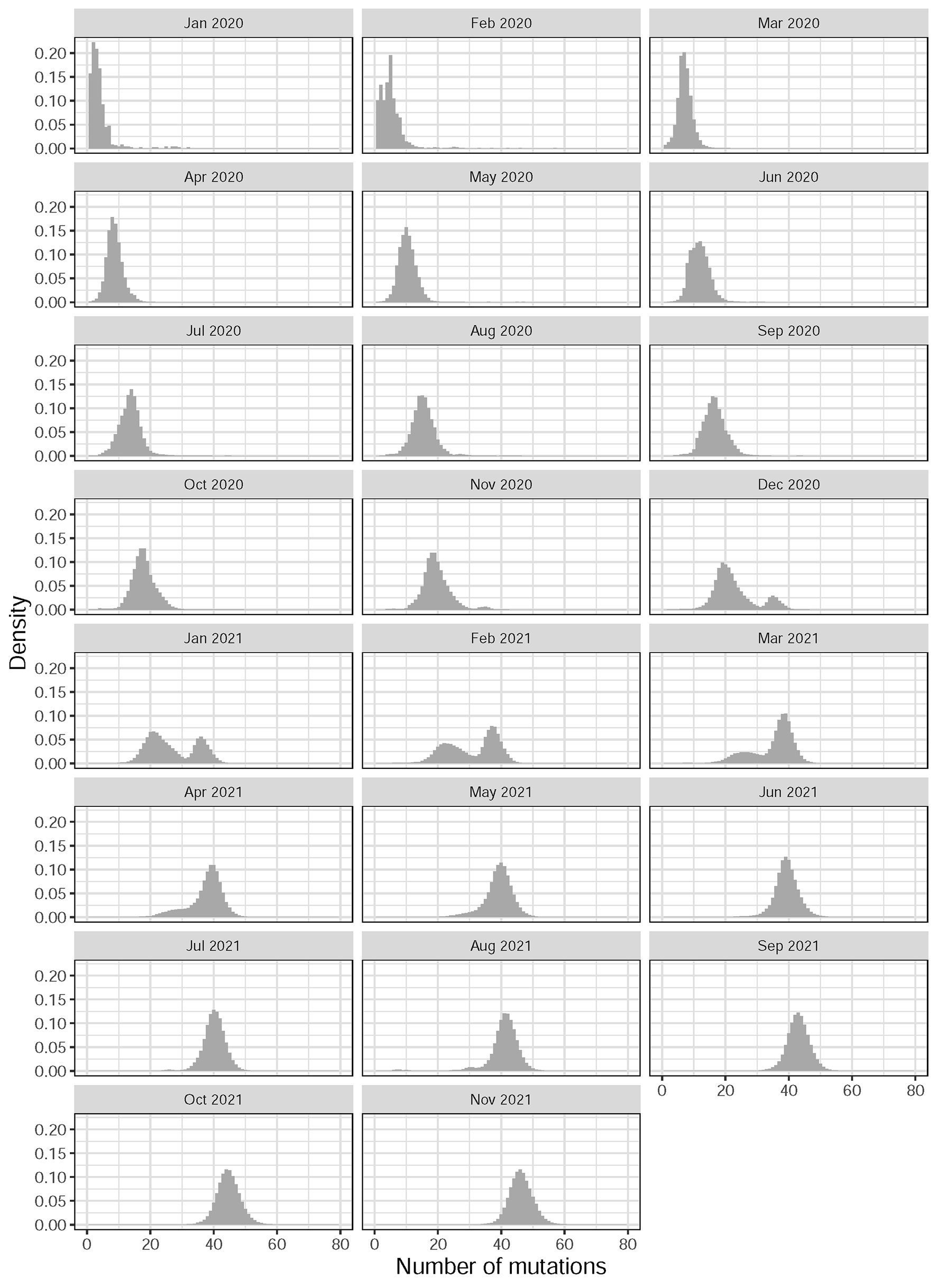

Supplement: Supplementary Figure 1 — Frequency distribution of mutational number of each SARS-CoV-2 genome at each sampling week. [file Image_1.TIF]

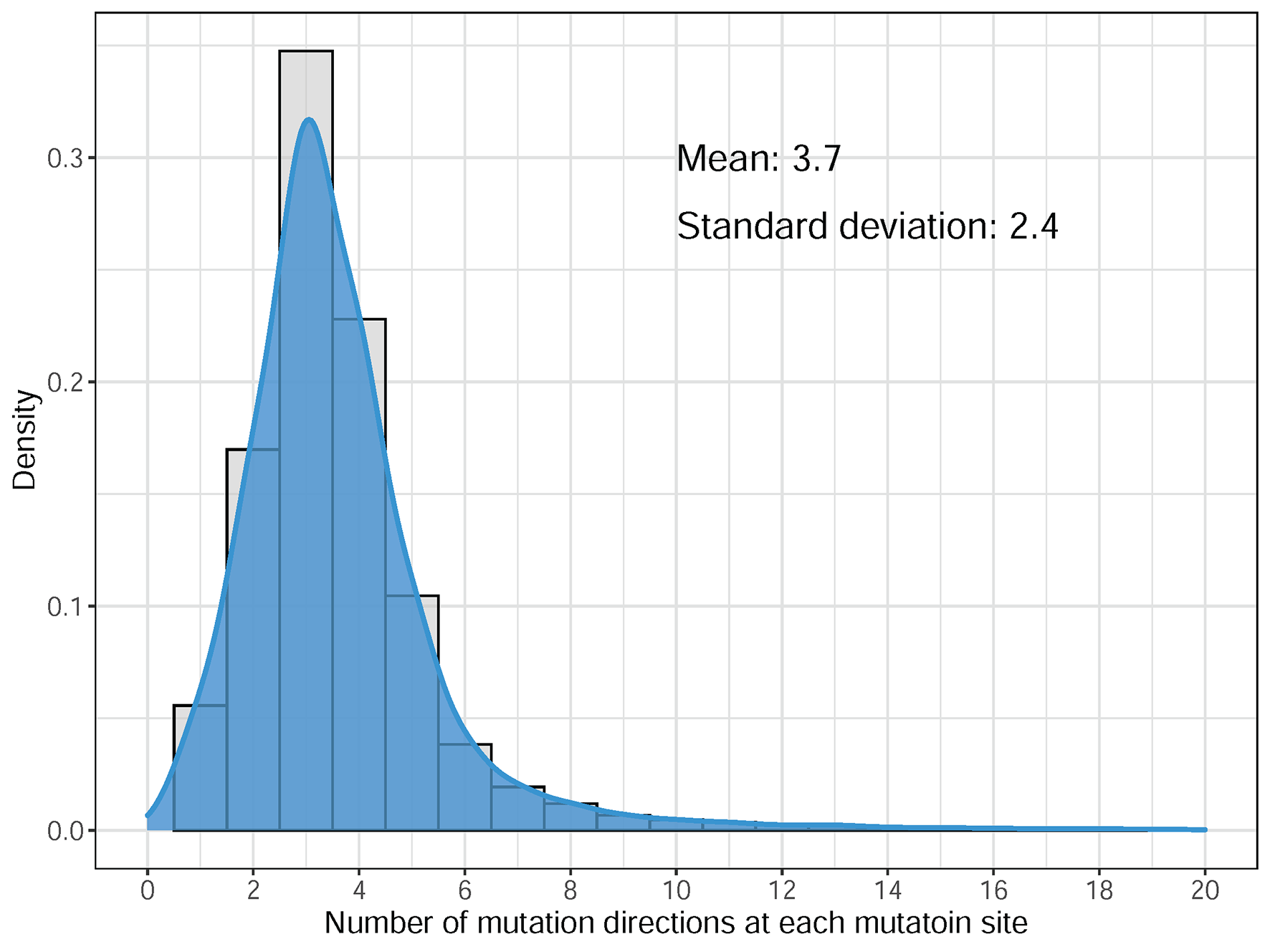

Supplement: Supplementary Figure 2 — Frequency distribution of number of mutation directions at each mutation sites. [file Image_2.TIF]

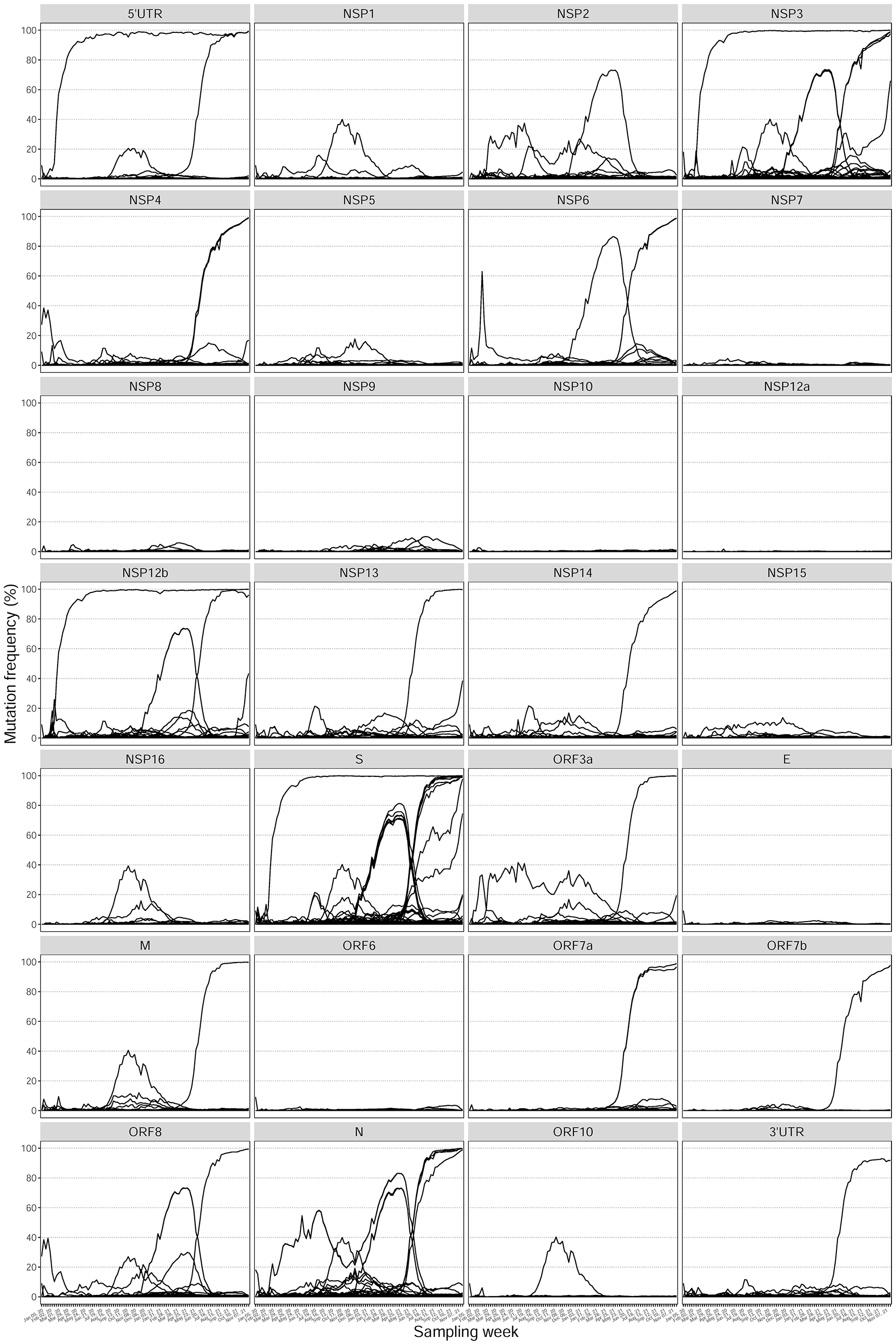

Supplement: Supplementary Figure 3 — Frequency trajectories of mutations by SARS-CoV-2 genome regions. UTR, Untranslated region; NSP, non-structural protein; S, Spike protein; ORF, Open reading frame; M, Membrane protein; N, Nucleocapsid protein; E, Envelope protein. [file Image_3.TIF]

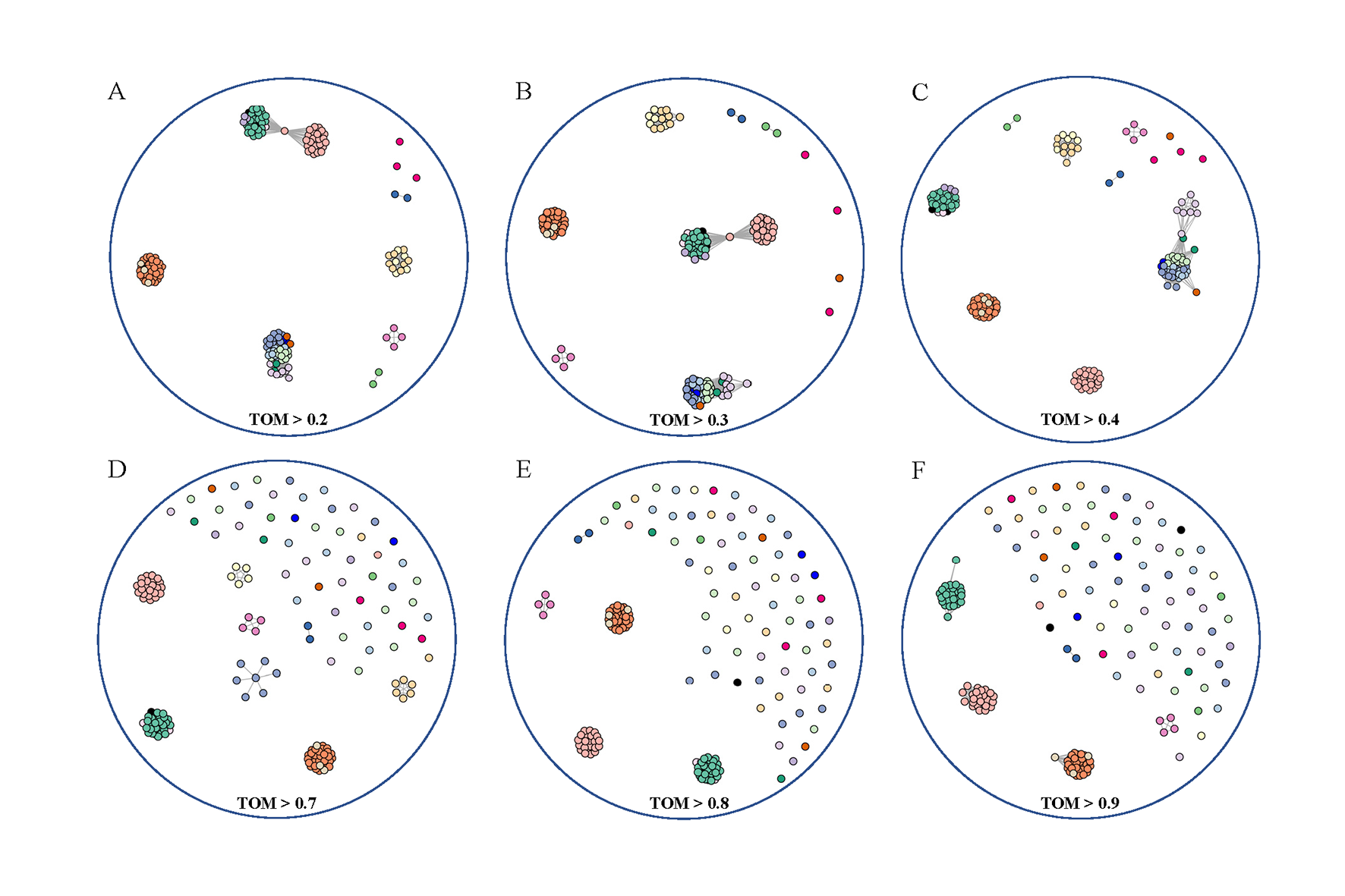

Supplement: Supplementary Figure 4 — Network graphs with different topological overlap cutoffs for identification of worldwide pandemic variants. [file Image_4.TIF]

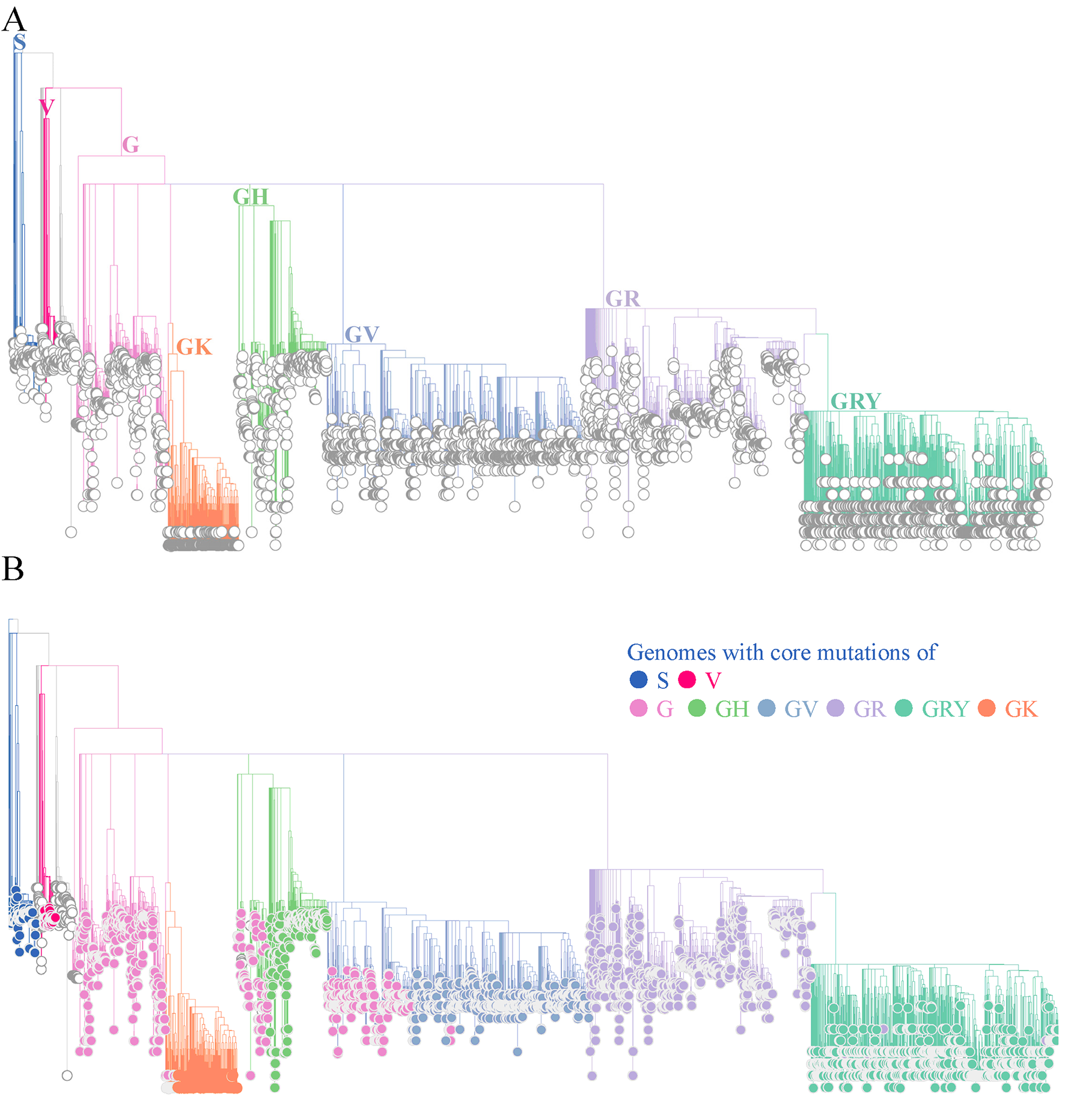

Supplement: Supplementary Figure 5 — The global SARS-CoV-2 phylogenic skeleton. (A) The SARS-CoV-2 phylogenic skeleton generated by the Nextstrain pipeline based on a random sample of the global phylogenic tree from the GISAID database, with the edges colored by the GISAID clade nomenclature system. (B) Comparison of the genome classification consistency between the expectation and those determined by the “core” mutations. [file Image_5.TIF]

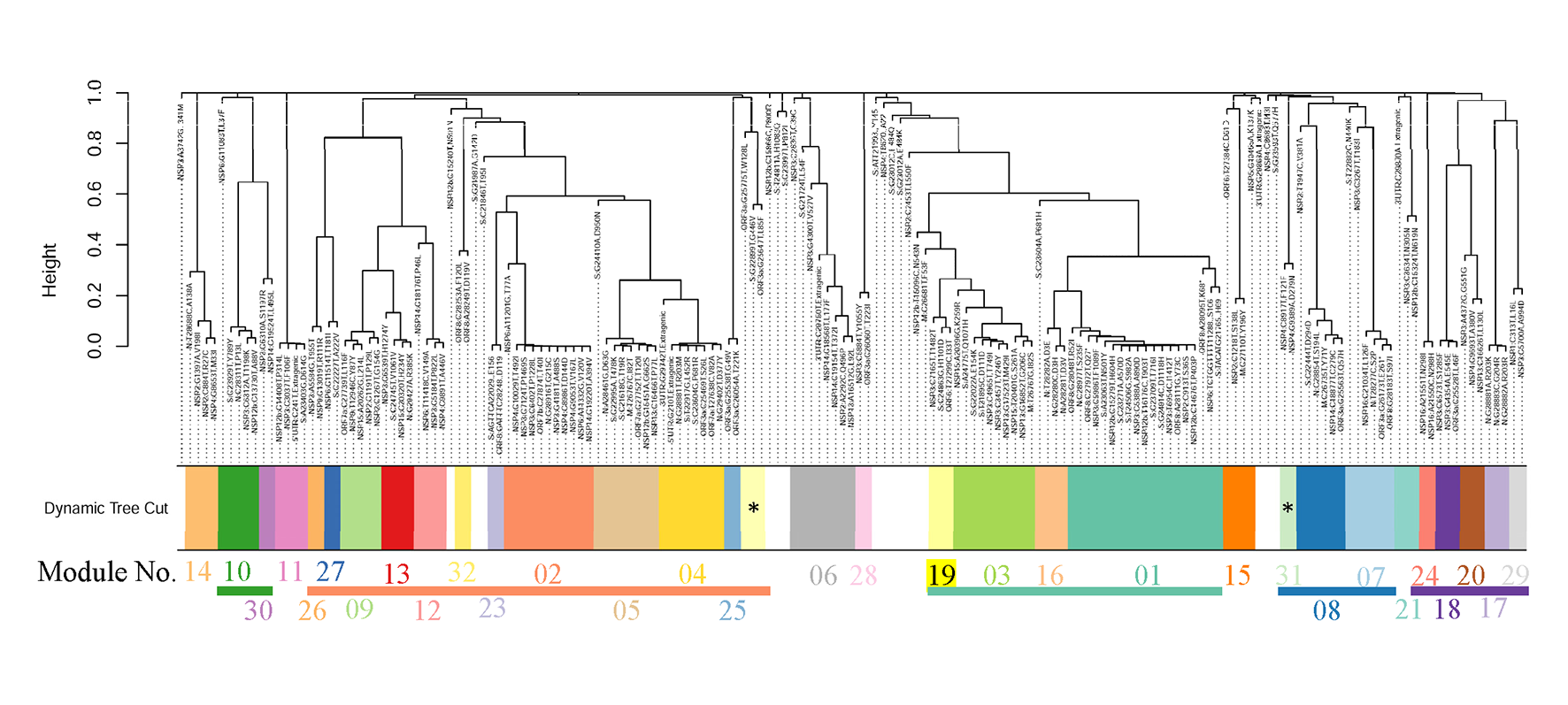

Supplement: Supplementary Figure 6 — Clustering dendrogram of 165 FTMs from India, with dissimilarity based on topological overlap. The module numbers were labeled and module clusters were highlighted with different colors. [file Image_6.TIF]

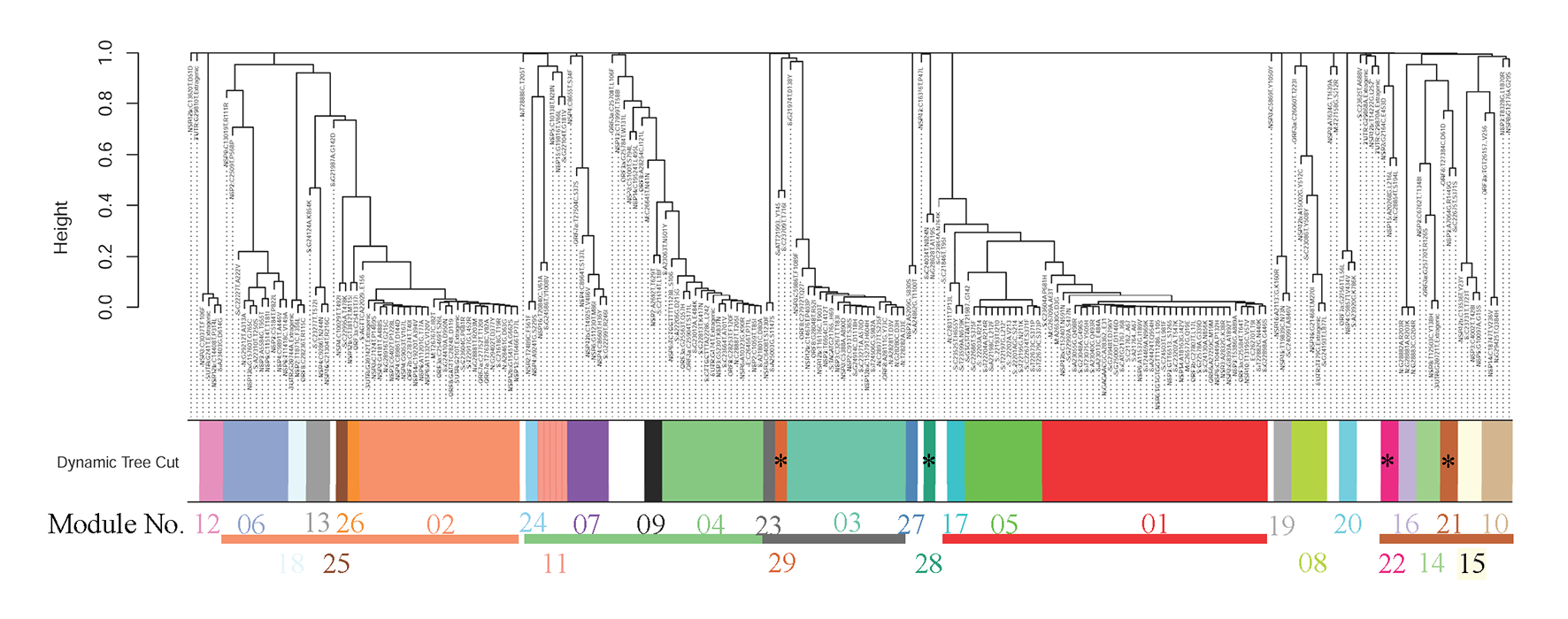

Supplement: Supplementary Figure 7 — Clustering dendrogram of 223 FTMs from South Africa, with dissimilarity based on topological overlap. The module numbers were labeled and module clusters were highlighted with different colors. [file Image_7.TIF]
